# Anxiety, Cognitive Biases, and Evaluative Conditioning: An Eye-Tracking Experiment

**DOI:** 10.5334/irsp.1205

**Published:** 2026-05-11

**Authors:** Anca Lazar, Marine Rougier, Jan De Houwer, Marco Perugini, Andrei Rusu, Florin Alin Sava

**Affiliations:** 1 Department of Psychology, West University of Timișoara, Romania; 2 Department of Experimental Clinical and Health Psychology, Ghent University, Belgium; 3 Department of Psychology, University of Milano-Bicocca, Italy

**Keywords:** evaluative conditioning, ambivalence, anxiety, cognitive bias, eye-tracking

## Abstract

Prior research revealed a correlation between individual differences in neuroticism (specifically, the component of trait anxiety) and evaluative conditioning (i.e., changes in attitude due to stimulus pairings). In two studies (*N* = 274 and *N* = 294), we examined which cognitive biases may explain this relation. In an evaluative conditioning procedure that included either clearly valenced (positive or negative) or ambivalent (positive and negative) stimuli, we used eye-tracking technology to measure attentional bias (dwell time within stimulus areas of interest), valence recollection tests to measure contingency awareness and memory bias, and adjective ratings to assess interpretation bias. In the second experiment, we additionally assessed attentional avoidance strategies. Correlation analyses did not reveal significant associations between any personality variable (neuroticism, anxiety, behavioral inhibition) and evaluative conditioning effects in either experiment. Implications are discussed in terms of methodological and theoretical contributions.

In everyday life, people are often confronted with mixed information. For instance, meeting a new co-worker may initially feel neutral, but if encounters with them are repeatedly accompanied by both positive outcomes (e.g., good teamwork) and negative ones (e.g., criticism), their presence can acquire a mixture of positive and negative evaluative associations—ones that individuals high in trait anxiety may be especially prone to interpret in a more negative way. Understanding how individuals manage to extract an overall evaluation based on mixed information is central to attitude and social cognition research. It may also help explain why, based on the same input, some individuals come to hold more negative evaluations than others. The present work focuses on evaluative conditioning (EC) as one pathway of attitude formation and investigates whether and how it is shaped by anxiety-related cognitive biases, especially when learning occurs in evaluatively ambivalent contexts.

## Evaluative Conditioning

EC refers to a change in attitude toward a neutral conditioned stimulus (CS) after pairing with a positive or negative unconditioned stimulus (US; De Houwer, Thomas, & Baeyens, 2001). Neutral CSs are usually liked more when repeatedly paired with positive (versus negative) USs.

From a theoretical perspective, EC is unlikely to be a mere automatic by-product of CS–US co-occurrence. The propositional account posits that EC arises when people encode and endorse propositions about CS–US relations (e.g., ‘this CS goes with that US’), a process assumed to require the attention and awareness of CS–US contingencies (De Houwer, 2018). Consistently, EC is disrupted when attention is drawn away from the pairings (Kattner, 2012), and it is stronger or only present when participants know or can remember which CS was paired with which US (Bar-Anan, De Houwer, & Nosek, 2010; Gast, Richter, & Benedict, 2025; Hofmann et al., 2010; Moran et al., 2023).

Most EC studies use clearly positive or negative USs consistently paired with neutral CSs, but in everyday life, the same CS may appear with both positive and negative stimuli, which makes ambivalent stimuli particularly relevant and ecologically valid. On the one hand, EC studies with mixed-valence stimuli confirmed that ambivalence can be conditioned but showed that contingency awareness plays a limited role (Glaser et al., 2018). Other work has shown that EC with conflicting valence information does not necessarily yield simple averaging effects; rather, the integration of positive and negative information depends on contextual and cognitive factors (Glaser & Walther, 2012). On the other hand, Weber et al. (2025) suggest that when a CS is paired with multiple USs of differing valence, the resulting evaluation reflects a weighted integration of valence cues even though negative information can exert disproportionate influence under some conditions. Bunghez et al. (2023) investigated the impact of individual differences on conditioning with ambivalent stimuli, suggesting that EC may depend not only on exposure but also on how mixed-valence information is attended, encoded, and interpreted depending on each individual’s perception, cognitive structure, and personality differences.

These findings converge on the idea that attention, memory, and interpretation biases are key levers of EC: (a) attentional bias determines to which elements of USs are attentional resources allocated to, (b) memory bias influences which CS–US pairings are retained and accessible later, and (c) interpretation bias shapes the overall meaning assigned to the learning situation (e.g., positive or negative). Such biases are conceptualized as systematic deviations from normative processing standards that may be functional in certain environments, for example, prioritizing threat-related information^1^ (Leung et al., 2022). In the present study, we investigate whether such cognitive biases may underlie systematic individual differences in EC.

## Neuroticism, Trait Anxiety, and EC

Early evidence suggests that personality differences may influence attention to CS without affecting the magnitude of the EC effect. More specifically, Fulcher et al. (2001) found that evaluative learning occurred similarly across neuroticism groups, yet high-neuroticism individuals showed greater attentional interference from negatively conditioned stimuli in probe detection tasks. This suggests that while trait anxiety may not moderate the formation of conditioned evaluations, it systematically biases subsequent attentional processing toward stimuli that have acquired negative valence, potentially through threat-detection mechanisms operating independently of the learning process itself.

Relatedly, mechanisms associated with anxiety may shape evaluative learning. For example, individual differences in emotion regulation moderate EC, with emotion dysregulation weakening positive conditioning and reappraisal strengthening it (Besson, Richetin, & Zerhouni, 2023), which suggests that difficulties in emotion regulation, common among individuals high in neuroticism, may help explain when personality traits relate to EC.

Several recent studies (e.g., Bunghez et al., 2023; Casini et al., 2023; Ingendahl & Vogel, 2023; Lazar et al., 2025; Vogel, Hütter, & Gebauer, 2019) indicate that neuroticism, trait anxiety, and related constructs can be linked to EC, but also suggest that the relation is modest and context-dependent. We reasoned that incorporating cognitive biases could help clarify this pattern. Hereafter, we present work showing that trait anxiety affects these biases. Specifically, we review evidence for the three cognitive biases most consistently associated with anxiety (Leung et al., 2022): attentional, memory, and interpretation bias.

### Attentional bias

Attention can be defined as a set of cognitive functions that prioritize certain stimuli for processing, given limited cognitive resources (Shechner et al., 2012). It is crucial because it gates the involvement of other processes such as memory and various forms of learning. Corbetta and Shulman (2002) distinguish between a top-down, goal-directed system driven by goals, expectations, and knowledge, and a bottom-up, stimulus-driven system that responds automatically to salient or unexpected events.

Anxiety is widely thought to bias these attentional processes (Cisler & Koster, 2010). According to Attentional Control Theory (Eysenck et al., 2007), anxiety reduces goal-directed attentional control and increases the influence of stimulus-driven processing, especially for threat-related stimuli. Anxiety is also proposed to impair inhibitory mechanisms needed to disengage from threat, and worry is seen as a key component that consumes limited attentional resources and working memory capacity. Thus, more anxious individuals are expected to orient more toward threat, whereas less anxious individuals may divert attention away from threatening information (Williams et al., 1988). Empirically, there is substantial evidence for anxiety-related attentional biases (Bar-Haim et al., 2007; Everaert, Duyck, & Koster, 2014; Marchetti et al., 2018). A meta-analysis of over 170 studies reported a moderate bias (*d* = 0.45) when threat stimuli were presented for 500 ms or less, indicating a robust early bias toward threat in anxious individuals compared to non-anxious individuals (Bar-Haim et al., 2007). Koster et al. (2006) similarly found an initial bias toward threat in high trait-anxious individuals, followed in some cases by later avoidance when stimuli were presented for longer, suggesting dynamic patterns of vigilance and avoidance. Consistent with this, Lommen, Engelhard, and van den Hout (2010) showed that individuals high in neuroticism or trait anxiety display a negative bias in processing ambiguous stimuli, often through avoidant behavior. Such strategies, including attentional deployment away from aversive cues, are common emotion regulation tools (Gross, 1998). Together, these findings imply that the influence of trait anxiety on attention allocation is likely moderated by avoidance tendencies.

Eye-tracking work provides converging evidence. A meta-analysis of eye-tracking studies reported a medium-sized attentional bias (*g* = 0.47) for anxious individuals, specific to negative stimuli (Armstrong & Olatunji, 2012). More recent work using neutral and negative images found that dwell time on negative pictures increased with higher trait anxiety, indicating that anxious individuals maintain attention longer on negative content (Veerapa et al., 2020).

### Memory bias

Memory bias can be defined as a tendency to remember certain types of information (e.g., threat-related stimuli) better than others, but also as a consistent, inaccurate recall of ambiguous information (e.g., in either a positive or negative manner). We note that the literature on memory bias in anxiety disorders is abundant but inconsistent. A meta-analysis comprising 171 studies on recall and recognition tasks using threatening stimuli concluded that the memory bias related to anxiety was significant only in free-recall tasks where explicit memory was involved (*d* = 0.32) (Herrera et al., 2017). Instead, for implicit memory bias, meta-analysis results showed no significant impact of anxiety (Mitte, 2008), indicating that memory bias is related to top-down higher-order processes. Their results indicated that the high-anxiety groups recalled more threatening stimuli and fewer positive ones than the low-anxiety groups. Moreover, when provided with ambiguous situations, more anxious people tend to interpret them in a more catastrophic manner, thus reflecting a negative memory bias. Such a tendency to remember threat-related pairings better could bias subsequent evaluations of CSs in EC procedures, especially when USs are mixed in valence.

### Situation interpretation bias

Interpretation bias is the tendency to resolve ambiguity by assigning a systematically positive or negative meaning to the situation as a whole. Research shows that more anxious individuals tend to interpret unclear situations more negatively (Leung et al., 2022).

In the present work, we examined whether participants’ appraisal of the experimental learning context itself might influence EC outcomes. After conditioning, participants rated the overall experimental situation using the Situation Five model (Ziegler, Horstmann, & Ziegler, 2019), which characterizes everyday contexts along dimensions including outcome expectancy, briskness, lack of stimuli, cognitive load, and physical and psychological load. We focused on descriptors related to cognitive, psychological, and physical load (e.g., ‘demanding,’ ‘threatening,’ ‘stressful’), as these map onto contexts relevant for negative interpretational tendencies. Because the perceived meaning of a situation predicts behavior within that context, we hypothesized that individuals high in neuroticism might appraise the situation more negatively, potentially moderating EC effects with ambivalent USs.

## Objectives

This study aims to shed light on the relation between personality and EC by examining the role of attentional, memory, and situation interpretation bias. We conducted two eye-tracking experiments using both ambivalent and classic monovalent (unambiguously positive and negative) USs. Our main pre-registered model focused on the ambivalent US condition because procedures with stimuli of mixed valence might best capture individual differences in cognitive biases. Nevertheless, including conditions for monovalent USs (positive and negative) allowed us to verify that our procedures produced significant standard EC effects (H1). As for the ratings of CSs paired with ambivalent USs, we hypothesized that the change in time (pre- versus post-conditioning) would correlate with neuroticism and the anxiety facet specifically (H2).

**Figure 1:**
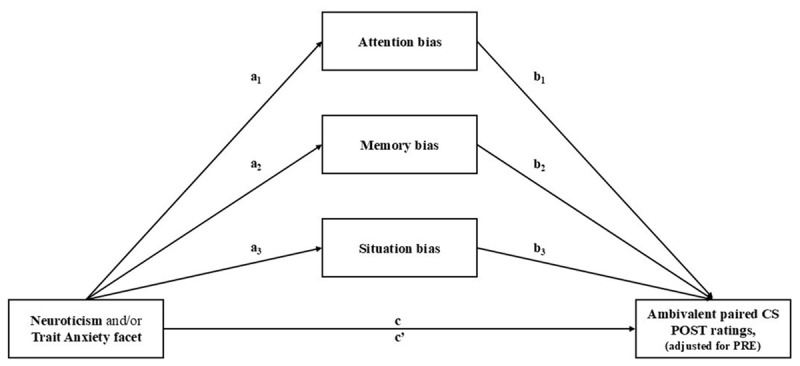
Parallel mediation model for the effect of neuroticism and/or trait anxiety on ambivalent EC.

Regarding the pre-registered mediation hypotheses involving the role of cognitive biases, we also aimed to investigate whether people with higher levels of neuroticism and trait anxiety were prone to a negative evaluative learning bias through a set of possible mediating factors: attention, memory, and situation interpretation bias. Therefore, we expected high-neurotic individuals to focus their attention on the negative elements of ambivalent USs (i.e., spend more time looking at them), which in turn should lead to a more negative rating of the paired CSs (H3.a). In terms of memory bias, we expected high-neurotic individuals to wrongly recall these CSs as being paired with a negative condition, ultimately leading to a more negative evaluation of those CSs (H3.b). As for the situation interpretation bias, we hypothesized that high-neurotic individuals would appraise the experimental situation more negatively, leading again to a more negative evaluation of the CSs paired with ambivalent USs (H3.c). The mediation model is presented below in Figure 1. Note, however, that the merits of these mediation analyses depend on a number of preconditions, such as whether there is a relation between the change in liking and the personality-related variables in the first place, or an indicator of a significant link via one of the proposed mediators. We report the results of the mediation models in the supplemental materials when the preconditions are not met.

## Experiment 1

### Methods

#### Design

We pre-registered the study rationale, hypotheses, methods, and planned analyses (https://aspredicted.org/n3wv-sp5h.pdf) and report the deviations in a document available at: https://osf.io/7kj9y/?view_only=472899933f524e7b835f939bf1aded5f. The results of Experiment 1 should be interpreted considering the methodological deviations (primarily concerning exclusion criteria and measurement decisions). Importantly, its core mediation hypotheses remained unchanged and informed procedural refinements implemented in Experiment 2. The experiment involved a 2 × 3 mixed design with a repeated factor (time of CS evaluation: before versus after conditioning) and a within factor (US valence: positive versus negative versus ambivalent).

#### Participants

To determine the sample needed to test our mediation models, we conducted a Monte Carlo power analysis for indirect effects (https://schoemanna.shinyapps.io/mc_power_med/). We set a target power of 0.8 for a parallel mediation model with three mediators, considering a correlation of *r* = .4 between the variables, therefore resulting in a necessary sample of 240 participants. Considering our exclusion criteria and the complexity of the model, we aimed for a higher sample, targeting around 300 participants. We managed to recruit 290 undergraduate students who participated in exchange for extra credit upon completion of both phases of the procedure. Participants confirmed by signing the consent form that they were over 18 years and met the inclusion criteria: no complex medical condition (e.g., strabismus, nystagmus) and no bi/trifocal or progressive glasses. These inclusion criteria were used to facilitate the calibration of the eye-tracking device.

We also pre-registered exclusion criteria to ensure we analyze qualitative and relevant data. First, in terms of variability in CS ratings, we eliminated 10 participants based on their pre-conditioning CS ratings, as they evaluated more than 50% of the CSs with extreme ratings (1 or 9), indicating a strong bias for the fractals used as materials. Regarding the post-conditioning ratings, we removed two participants who evaluated more than 75% of the CSs with the same rating, indicating a disengaged attitude in the experiment post-conditioning. Secondly, we pre-registered a criterion related to contingency awareness, but it was not applied, with justifications provided in the deviation document uploaded on OSF. We also decided to add another criterion that we did not foresee and eliminated four outliers, considering a threshold of +/–3 SD in the average attention dwell time. Focusing entirely on either the CS or US prevents individuals from encoding the other stimulus and indicates disengagement from the participants in the conditioning procedure. This is in line with previous findings suggesting that focusing attention on one stimulus is not sufficient for EC to occur, while attention to the pairing is crucial to foster contingency awareness (Kattner, 2012). This left us with a final sample of 274 participants (69% females; *M_age_* = 27.13, *SD_age_* = 9.83).

#### Materials

##### Instruments

We used a 48-item neuroticism subscale of NEO-PI-R (McCrae & Costa, 1992) including six facets: anxiety, depression, anger, timidity, impulsivity, and vulnerability. Items were evaluated on a 5-point Likert scale, with a higher score indicating higher levels of neuroticism. To assess participants’ interpretation of the situation during the conditioning experiment, we used a set of attributes extracted from the Situation Five taxonomy (Ziegler, Horstmann, & Ziegler, 2019), referring to cognitive load (unpleasant, boring, chaotic, challenging, mentally stimulating, and demanding) as well as psychological and physical load (tensed, strenuous, laborious, annoying, depressing, burdensome, and physically demanding). Participants indicated to what extent each of these attributes described the way they perceived the experiment situation using a 5-point scale (from 1 = *not at all* to 5 = *very much*).

##### Stimuli

**Figure 2:**
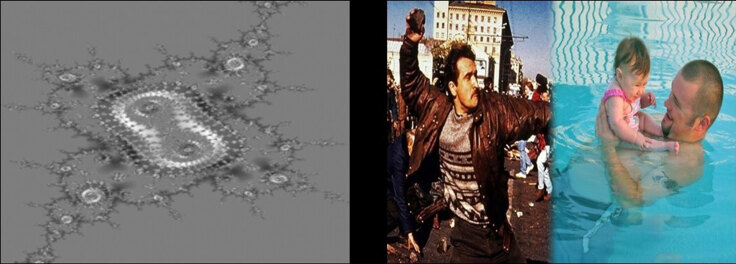
Example of a CS–US pairing for the ambivalent condition. *Note*. An ambivalent pairing shows the CS on the left and the US on the right, composed of two elements of opposite valence merged together. Monovalent USs comprise two elements of the same valence.

For the conditioning experiment, we used eight CSs paired with three US valence conditions, including two positive USs, two negative USs, and four ambivalent USs. For CSs, we used abstract images of computer-generated, gray-scale fractals that were also used in previous studies on EC and anxiety (Bunghez et al., 2023; Casini et al., 2023). Each of the eight USs was a compound image of two merged pictures (as in Glaser et al., 2018; Bunghez et al., 2023) and retrieved from International Affective Picture System (IAPS) (Lang, Bradley, & Cuthbert, 2005). For monovalent conditions, both pictures conveyed the same valence, while ambivalent USs consisted of a positive and negative image, both chosen based on a similar degree of arousal, dominance, and valence extremity to keep both sides balanced. Figure 2 provides an example of a stimulus pairing for the ambivalent condition.

It is worth noting that the negative USs used in Experiment 1 targeted general negative valence (e.g., images of threat, disgust, or sadness) rather than a single, narrowly defined emotional category, although some past research on anxiety has focused specifically on threat-relevant stimuli, particularly in social contexts (e.g., Bar-Haim et al., 2007). The deliberate use of heterogeneous negative stimuli here serves an important purpose: it allows findings to generalize across the range of aversive content that individuals with elevated trait anxiety may encounter, rather than being tied to one specific emotional signal.

Additionally, to counterbalance the positioning of the two images included in the compound ambivalent stimulus, we switched sides for two of the mixed valence stimuli. Hence, two out of four ambivalent USs have positive pictures on the left, and the other two have negative pictures on the left. Furthermore, we randomized the CS–US pairings across participants by preparing eight groups, ensuring each CS was paired with each of the eight USs.

##### Attention

To evaluate biases during conditioning, we defined three Areas of Interest (AOIs): the CS and the two sides of the ambivalent US. Among several eye-tracking indices, we focused on total dwell time (Duration of Visits; DOV), reflecting the total time (ms) spent on each AOI; for exploratory purposes, we also recorded the first AOI accessed to capture initial attentional biases. DOV values were averaged across the eight trials per pairing and used to compute a net attention score (DOVnet) for ambivalent USs (dwell time on positive – dwell time on negative elements), such that positive values indicate a positive bias and negative values a negative bias. For monovalent conditions, we applied the same logic (DOVnetM = dwell time on positive USs – dwell time on negative USs). The first AOI variable was calculated as the net number of trials (out of 32 ambivalent trials) in which participants first fixated on the positive versus the negative element.

##### Memory

To assess contingency awareness (*totVA*), that is, whether participants could recall the valence of the US paired with each CS, the eight fractals were presented again after post-evaluation, and participants indicated whether each had been paired with a positive, negative, or ambivalent US. For ambivalent trials, this also allowed us to examine potential memory bias (*mmb*) computed as the difference between the number of ambivalent pairings wrongly recalled as positive versus negative, with positive values indicating a positive bias and negative values a negative bias.

#### Procedure

Data collection followed a two-step procedure. First, participants completed an online survey (consent, demographics, and individual-differences questionnaires). Second, they attended an individual lab session for the conditioning experiment using a screen-based eye tracker (Tobii Pro Fusion). After calibration to ensure appropriate viewing distance (~68 cm) and acceptable data quality (<15% data loss), participants completed the following tasks using a numerical keypad.

Each CS was first presented alone for 2000 ms, followed by pre-conditioning likeability ratings (from 1 = *strong dislike* to 9 = *strong like*). Participants were then randomly assigned to one of eight CS–US pairing groups and instructed to look at both stimuli, as they would later be questioned about them. During conditioning, each pair appeared eight times (64 trials total) for 3500 ms on a black background; trials were separated by a 500 ms fixation cross requiring gaze recentering. After conditioning, participants again rated CS likeability and completed a contingency awareness task, indicating for each CS whether it had been paired with a positive, negative, or ambivalent US, or selecting ‘do not remember’ (to discourage guessing). Finally, we assessed situation perception bias.

### Results

#### Preliminary analysis

We performed analyses using R software (version 4.4.1). Descriptive statistics are presented in the supplemental materials (Table S1.1). Reliability for the neuroticism measure (NEO-PI-R) was high (*α* = .93), and for each of its individual facets, it was good (ranging from *α* = .73 to *α* = .83), except for the impulsiveness facet (*α* = .66). The dataset and R script are available at: https://osf.io/7kj9y/?view_only=472899933f524e7b835f939bf1aded5f.

First, we computed the mean CS score per condition (positive, negative, and ambivalent) and timepoint (pre and post), together with the EC effect (calculated as the difference in average post rating for CSs positively paired versus those negatively paired). For the ambivalent condition, we considered the change score as we did not have a neutral condition to compare against.

**Figure 3:**
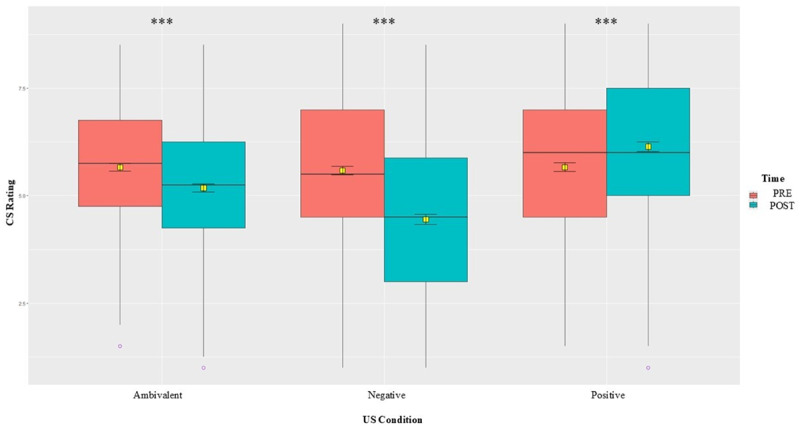
Box-plot with average pre/post CS ratings by US condition. *Note*. Means represented by yellow squares with error bars indicating the 95% confidence interval (CI). Horizontal lines and circles visualize the medians and outliers. Significance levels: ***(*p* < .001), **(*p* < .01), *(*p* < .05), *ns* (not significant).

We first tested the interaction between time and US valence on CS ratings by means of a mixed ANOVA. Results presented in Table S1.2 show a significant interaction between time and US condition of *F* (2,445) = 58.62, *p* < .001, *η²* = .18, indicating that EC has indeed occurred. Bonferroni-corrected pairwise comparisons by time and US condition (Tables S1.3 and S1.4) show a significant change in time for all three US conditions, but the largest change is shown for the CSs paired with negative USs. There were no significant differences between the CSs’ pre-ratings, but after conditioning, the differences between conditions were significant, especially between the standard monovalent conditions (positive versus negative) with a medium effect of Cohen’s *d* = 0.60, 95% CI [0.47, 0.73], *p* < .001, thus confirming our first hypothesis (H1). A visual representation of the changes in CS ratings over time and between US conditions (Figure 3) shows that both negative and ambivalent mean ratings decreased after conditioning, while the positive slightly increased. We also tested the interaction of CS evaluations with US valence (positive versus negative) using mixed model analysis to better account for variation at both participant and stimulus (CS) levels. The overall pattern of results (shown in Table S1.5) remains unchanged.

#### Correlations

**Figure 4:**
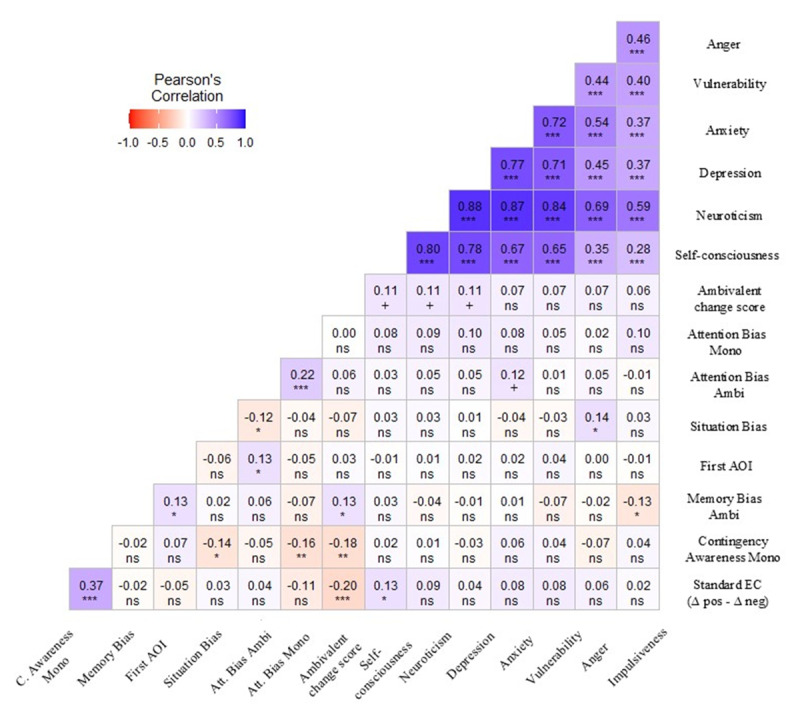
Pearson correlations between the variables of interest. *Note*. Significance levels: ***(*p* < .001), **(*p* < .01), *(*p* < .05), +(*p* < .10), *ns* (not significant).

In Figure 4 below, we verified the correlations between our main dependent variables (change over time in CS ratings paired with ambivalent USs), as well as the standard EC effect (calculated as the change in time for positive versus negative conditions), neuroticism (along with its facets), and the three proposed mediators (attention, memory, and situation interpretation bias). Neuroticism facets were strongly intercorrelated, but their associations with the change in ratings for CSs paired with ambivalent USs were small and non-significant, including overall neuroticism (*r* = .11, *p* = .075, 95% CI [–0.01, 0.22]) and the anxiety facet (*r* = .07, *p* = .262, 95% CI [–0.05, 0.19]), providing no support for H2. Among the proposed biases, anger was linked to a more negative situation interpretation bias, impulsiveness to a more negative memory bias, and anxiety showed a trend toward a positive association with attention bias, indicating that as anxiety levels increase, so does the tendency to direct attention to positive elements within ambivalent CSs. Changes in ratings for CSs paired with ambivalent USs were positively related to memory bias and to the first AOI accessed, such that initially fixating positive (versus negative) elements was associated with recalling the CS as paired with a positive (versus negative) condition; attention bias (DOVnet) was also related to the first AOI and to a more negative situation interpretation bias. For standard EC with monovalent USs, effects were positively associated with self-consciousness and, more strongly, with contingency awareness.

#### Effect of the position of ambivalent elements on attention metrics

Looking at the attention data, we observed a bias toward the ambivalent element positioned closest to the center—considering the three AOIs within ambivalent conditions (the CS on the left, one of the ambivalent US elements on the right, and the other in the center-right). Therefore, we conducted a within-participants mixed model analysis testing whether the valence of the center element of ambivalent USs (coded with positive = +0.5 and negative = –0.5) predicted the direction of the attention bias for each participant (DOVnet: measured as the average difference in milliseconds between the time spent focusing on the two sides of the ambivalent USs). Results showed a strong relationship *(β* = 978.27, 95% CI [925.9;1030.65], *SE* = 26.69, *t* = 36.65, *p* < .001), indicating a structural position bias, as indeed the valence of the centered element interferes with attention.

#### Mediations

Considering the results presented above, which indicate a structural bias that compromises the accurate measurement of the attention bias, corroborated with the lack of correlation between the change in CS ratings and neuroticism or trait anxiety, the merits of mediation analyses are limited. Yet, as we pre-registered a model with three parallel mediators, we included the results in the supplemental materials, both using neuroticism (Figures S1.1) and trait anxiety (Figure S1.2) as main predictors and controlling for the pre-evaluations as covariate (PROCESS model 4; Hayes, 2022). Both models indicated that memory bias predicts the change in liking of CSs paired with ambivalent content (*b_2_* = 0.17, *p* = .003), but it is not linked to the personality variables. Anxiety appeared to positively predict the attention bias (a*_1_* = 4.59, *p* = .046), such that individuals scoring higher on the anxiety measure also focused more on positive elements within ambivalent USs. The third mediator on interpretation bias was not significant. Importantly, we refrain from interpreting these results further in relation to H3 due to the strong confounding effect of the structural bias. Note that we also report these models applying all the pre-registered exclusion criteria (Figures S1.3 and S1.4).

To maximize the value of the eye-tracking data and address position bias in ambivalent stimuli, we conducted an exploratory analysis in which ambivalent USs were recoded by the valence of the centrally positioned element. In this alternative framework, purely positive/negative USs produced stronger EC effects than ambivalent USs, with slightly larger differences for positive than negative conditions. Full details are reported in the supplemental materials (Tables S1.6–S1.8).

### Discussion

This first study examined how EC relates to personality differences in neuroticism and its anxiety facet, and whether this link is explained by cognitive biases. Preliminary analyses confirmed that the conditioning procedure produced a reliable EC effect, supporting H1. Across time, CS ratings increased slightly in the positive condition and decreased in the negative and ambivalent conditions, with ambivalent USs overall functioning similarly to negative USs. However, changes in liking for ambivalently paired CSs did not correlate significantly with either neuroticism or the anxiety facet. Hence, H2 was not supported. Among the bias measures, we observed a small positive association between memory bias (recalling ambivalent pairings as more positive versus negative) and changes in liking, and a non-significant trend for more anxious individuals to fixate longer on positive elements.

Interpretation of attentional effects was compromised by a strong structural position bias: participants tended to fixate near the screen center, so the valence of the centrally located element largely drove both gaze and CS evaluations. Given this bias and the lack of association between personality and ambivalent EC, we did not discuss the mediation models relevant for H3. To make use of the eye-tracking data, we conducted an exploratory analysis comparing EC effects from purely positive/negative USs to ambivalent USs recoded by the valence of the central element. USs with clear, unambiguous valence produced significantly stronger EC effects than ambivalent USs with a peripheral opposite-valence element. These findings, together with limitations in US positioning and content heterogeneity, motivated a follow-up experiment with improved, more controlled ambivalent stimuli to re-examine the mediation model without the structural position bias.

## Experiment 2

Building on these findings and limitations, Experiment 2 was designed to refine both the methodological and conceptual aspects of our model. Methodologically, we modified the structure of the ambivalent stimuli by placing the CS in the center and the positive and negative elements on the sides to reduce potential position biases observed previously. We also used more homogeneous US content, two faces displaying different emotional expressions (happy versus angry), to limit variability in arousal and complexity and thereby improve ecological validity and control for stimulus heterogeneity.

Conceptually, we expanded the model by including experiential avoidance and behavioral inhibition (BIS) as additional individual-difference variables. Experiential avoidance was added as a potential moderator based on patterns indicated in Experiment 1 suggesting that more anxious individuals may look longer at positive elements within ambivalent USs, consistent with avoidance-related attentional mechanisms and prior work linking anxiety to biased attention toward or away from threatening information (Bar-Haim et al., 2007; Koster et al., 2006). In addition to the previously reported three hypotheses, we also tested whether individuals high in both trait anxiety and experiential avoidance focused more on the positive elements of ambivalent USs (H4). BIS was included as an alternative personality predictor, given its overlap with anxiety and its role in sensitivity to negative cues (Carver & White, 1994), as well as recent findings showing a marginal association between evaluative conditioning and BIS but not neuroticism or its anxiety facet (Lazar et al., 2025). We dropped situation interpretation bias because it did not correlate with our main variables and added unnecessary complexity.

### Method

#### Design

Experiment 2 also involved the same 2 × 3 mixed design as in Experiment 1. The pre-registration is available at: https://osf.io/azynk/?view_only=1f25dbade0f94f6db65f0be61d2abcd0.

#### Participants

We used the same sampling and exclusion procedure as described in Experiment 1. A total of 304 participants completed this study; however, seven participants were excluded due to biased pre-conditioning CS ratings, and one was eliminated due to lack of variability in CS ratings post-conditioning. As previously mentioned, the pre-registered exclusion criterion related to contingency awareness was not applied, and two participants were outliers based on excessive dwell time on one of the stimuli. Therefore, our final sample consisted of *N* = 294 (70% females, *M_age_* = 22.48, *SD_age_* = 6.27).

#### Materials

To assess individual differences potentially influencing EC and its underlying mechanisms, we used three self-report measures: trait anxiety (10-item IPIP-NEO anxiety subscale; Johnson, 2014), BIS (7-item BIS scale from the BIS/BAS; Carver & White, 1994), and experiential avoidance (15-item BEAQ; Gámez et al., 2014). Participants responded on 5-point, 4-point, and 6-point Likert scales, respectively, with higher scores indicating higher trait anxiety, BIS, and experiential avoidance.

**Figure 5:**
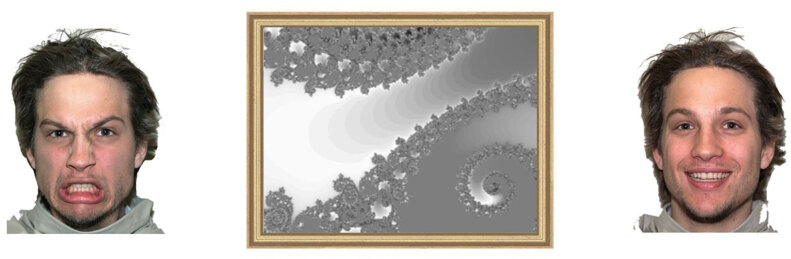
Example of CS–US pairing for the ambivalent condition. *Note*. Ambivalent stimuli consisted of a happy and an angry portrait placed on each side of a centered fractal as CS. Monovalent stimuli comprised two portraits expressing the same emotion.

As content material for the eight CSs, we used grayscale fractals as in the previous experiment, but for Uss, we used male portraits retrieved from the Nim Stim database (Tottenham et al., 2009) with different face expressions denoting positive (happy) or negative (angry) emotions, as presented below in Figure 5. Experiment 2 narrowed the stimulus set to only angry emotions, a well-validated category of socially threatening stimuli particularly relevant to trait anxiety (e.g., Bar-Haim et al., 2007), to test whether the null effect from Experiment 1 would persist under conditions theoretically more conducive to anxiety-related moderation. To mitigate the risk of a left-right processing bias, we counterbalanced the position of the two US elements; therefore, two of the four ambivalent USs had happy faces on the left, while the other two had angry portraits on the left.

#### Procedure

The procedure followed the same steps as in the previous experiment, with demographics and personality questionnaires (anxiety, behavioral inhibition, and experiential avoidance) administered online, followed by the conditioning procedure using the eye-tracker, although there were a few minor changes. In the pre-evaluation phase, we included four distractor fractals to mitigate a potential mere-exposure effect, which is the increase in liking of a stimulus due to its repeated exposure. We concluded with a short debriefing session right after evaluating awareness.

### Results

#### Preliminary analysis

Data analysis and variable computation were similar to the previous experiment and the reliability coefficients for IPIP-NEO (*α* = .89), BIS (*α* = .79), and BEAQ (*α* = .85) were all acceptable. The descriptive statistics for the main variables are available in Table S2.1 in the supplemental materials. The dataset and R script are available at: https://osf.io/7kj9y/?view_only=472899933f524e7b835f939bf1aded5f.

**Figure 6:**
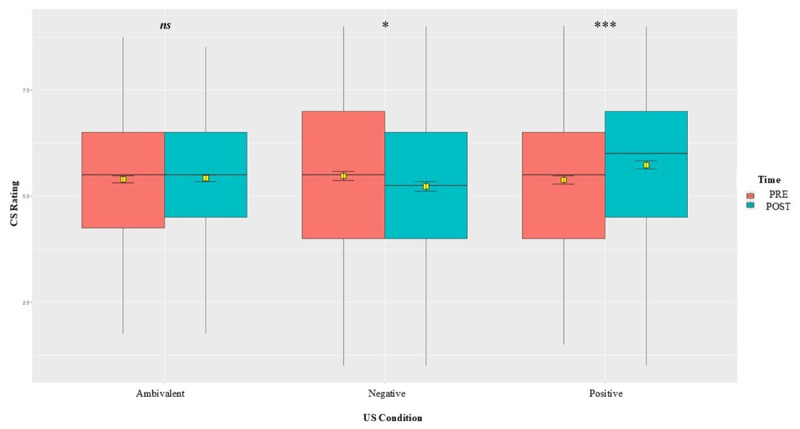
Box-plot with average pre/post CS ratings by US condition. *Note*. Means represented by yellow squares with error bars indicating the 95% CI. Horizontal lines and circles visualize the medians and outliers. Significance levels: ***(*p* < .001), **(*p* < .01), *(*p* < .05), *ns* (not significant).

To determine whether there are any significant changes in CS ratings due to conditioning, we performed a 2 × 3 mixed ANOVA with time (pre versus post) and US condition (positive versus negative versus ambivalent) as within factors. Results in the supplemental materials (Table S2.2) indicated a significant interaction effect between time and condition of *F* (2,540) = 13.42, *p* < .001, η² = .04. Figure 6 below, along with Bonferroni-corrected pairwise comparisons by time (Table S2.3), showed a significant increase in time for positive conditions, and a slight decrease for the negative conditions, but none for the ambivalent. Compared by condition, results (Table S2.4) also showed a significant difference between the post ratings of CSs paired with the positive condition compared against ambivalent and stronger against the negative condition (a small effect of Cohen’s *d* = 0.22, 95% CI [0.11, 0.34], *p* < .001), but none between the other two against each other. The significant change in liking of stimuli paired with positive and negative USs supports that the conditioning experiment was successful and confirms our first preliminary hypothesis (H1). We also tested the interaction of CS evaluations with US valence (positive versus negative) using mixed model analysis to better account for variation at both stimulus and individual levels. The overall pattern of results (shown in Table S2.5) remains unchanged.

#### Correlations

**Figure 7:**
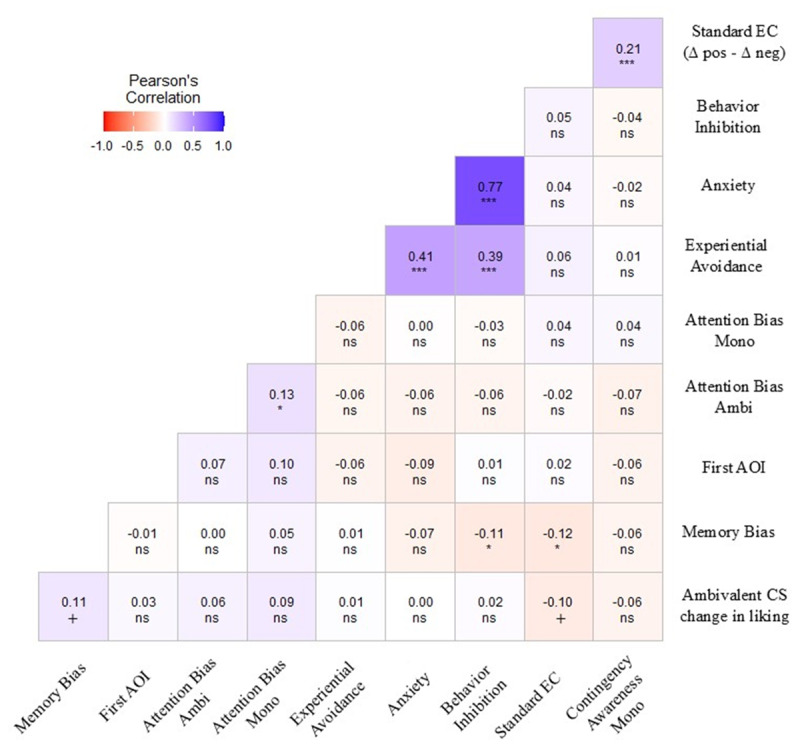
Pearson correlations between variables of interest. *Note*. Significance levels: ***(*p* < .001), **(*p* < .01), *(*p* < .05), +(*p* < .10), *ns* (not significant).

Although participants showed no overall change in CS ratings in the ambivalent condition, such null effects may mask opposing trends depending on personality and attentional strategies (e.g., anxious individuals focusing or avoiding negative elements). Correlational analyses between the change in liking for CSs paired with ambivalent USs and individual-difference variables are shown in Figure 7. Neither trait anxiety nor BIS was significantly related to the change in liking, so H2 was not supported. As expected, trait anxiety and BIS were strongly correlated, and both were moderately associated with experiential avoidance. Memory bias showed a small negative correlation with BIS (*r* = –.11, *p* = .049, 95% CI [–0.23, –0.00]) but was only marginally related to the change in liking (*r* = .11, *p* = .065, 95% CI [–0.01, 0.22]). The first AOI accessed did not correlate with any key variables and was not considered further. For the standard EC effect, correlations again indicated a robust association with contingency awareness.

#### Moderated mediation

Despite the lack of significant direct effects, we proceeded to test our pre-registered mediation model and additionally reran it using BIS as a predictor due to the slight trends resulting from the correlations. This suggested memory bias was linked to both personality and the change in liking, indicating a potential full mediation path. We examined whether trait anxiety (or BIS) explains evaluations of CSs paired with ambivalent USs via two potential mediators (attention and memory biases), and whether these paths are moderated by experiential avoidance, controlling for the pre-evaluations as covariate (PROCESS model 7; Hayes, 2022). Nevertheless, we acknowledge the limited merit of these mediation analyses when correlations are weak and marginally significant.

Model results are presented in the supplemental materials (Figure S2.1), where anxiety showed no direct effect on the change in liking, nor on attentional bias, so H3.a was not supported. However, experiential avoidance significantly moderated the effect of anxiety on attentional bias *(R²* = .02, F (1,289) = 5.82, *p* = .016), but not in the predicted direction (H4): simple slopes analysis (Figure S2.2 in the supplemental materials) indicated that higher anxiety was associated with longer dwell time on angry faces among participants high in avoidance. The moderation of anxiety on memory bias via avoidance was very small and not significant. There was no evidence for the hypothesized indirect effects (H3.a, H3.b), but memory bias had a direct positive effect on CS ratings (*b_2_* = 0.14, *p* = .006).

Repeating the model with BIS as the predictor (Figure S2.3) yielded similar results: neither the moderation by avoidance nor the indirect path via attentional bias was significant. However, we did observe a small association between BIS and memory bias *(a_2_* = –0.05, *p* = .023) and a positive effect of memory bias on CS ratings (*b_2_* = 0.15, p = .005), consistent with a weak indirect pathway from BIS to CS evaluations through memory bias. This offers only partial support for H3.b and should be interpreted cautiously, given the absence of a significant direct link between personality and change in CS liking.

We also tested the models applying all the pre-registered criteria using both anxiety (Figures S2.4 and S2.5 in the supplemental materials) and BIS (Figure S2.6) as predictors. Results were similar to those described above.

### Discussion

This second study aimed to clarify the relationship between trait anxiety and EC in ambivalent contexts by examining two cognitive biases (attention and memory) and the moderating role of experiential avoidance. We improved the ambivalent stimulus design and added a self-report measure of experiential avoidance to test whether it moderates the effect of anxiety on attention.

A 2 × 3 mixed ANOVA (time × US condition) showed a significant interaction. Pairwise comparisons indicated significant changes over time only for the positive and negative US conditions, but not for ambivalent USs. Compared to Experiment 1, monovalent USs produced a lower but significant standard EC effect, confirming H1. Moreover, ambivalent USs now behaved more like neutral stimuli. Correlations did not show a significant association between the change in liking for ambivalent CSs and anxiety or BIS, providing no support for H2. Yet higher BIS was linked to a more negative memory bias (ambivalent CSs more often recalled as paired with negative USs), and there was a near-significant trend whereby more positive (versus negative) recall of ambivalent pairings predicted more positive (versus negative) CS ratings. Although these trends were low, they indicated a potential indirect mediation path.

We therefore tested a moderated mediation model including attention and memory bias as mediators and experiential avoidance as a moderator. Avoidance significantly moderated the relation between anxiety and attention, but not in the direction hypothesized in H4. Specifically, findings indicated that with increasing anxiety, highly avoidant participants dwelled more on angry faces, contradicting the attentional disengagement strategy (Bar-Haim et al., 2007). Mediation paths via attention and memory did not support our hypotheses H3.a and H3.b, but memory bias was directly related to CS ratings: ambivalent CSs recalled as paired with positive (negative) USs were rated more positively (negatively). For BIS, we observed a small, marginally significant association with memory bias, such that higher inhibition predicted slightly more negative recall and, in turn, more negative CS evaluations.

## General Discussion

The present research examined possible processes underlying the EC effect, more specifically how anxiety-related biases could impact these processes. Building on Bunghez et al. (2023), who found that more neurotic individuals evaluated CSs paired with ambivalent USs more negatively, we tested whether such effects can be explained by well-known cognitive biases, such as: attention, memory, and situation interpretation. In two experiments, we found reliable EC effects for clearly valenced (monovalent) USs and mixed effects for ambivalent USs. However, our studies provided little evidence that anxiety-related traits systematically shape evaluations, because we did not find clear correlations with (ambivalent or monovalent) EC in the first place. These findings align with recent work reporting that these effects are small and inconsistent, often depending on sample size, applied methods, and statistical tests (Ingendahl & Vogel, 2023). A comprehensive summary table of all results from both experiments is available in the supplemental materials (Table S3).

A first implication of our findings concerns attitude research. In both studies, monovalent pairings produced standard EC effects that were largely independent of individual differences. In tightly controlled EC paradigms with repeated CS–US exposure and easily detectable contingencies, there may be limited scope for anxiety-related traits to influence evaluative learning. In this case, EC-based attitude formation appears driven primarily by the structure of the learning procedure rather than by who the learner is. We also acknowledge that mere-exposure effects, whereby repeated presentation alone can enhance liking (Bornstein & Craver-Lemley, 2017), may also contribute to the observed preference changes.

Regarding ambivalent EC, the differential pattern of ambivalent CS evaluations across experiments warrants consideration (negativity bias in Experiment 1, neutral in Experiment 2). This divergence in the results of our two studies may reflect differences in contingency awareness (participants were 5% more aware of ambivalent associations in Experiment 2) and stimulus presentation format (with Experiment 2’s bilateral US positioning potentially promoting more balanced averaging of positive and negative components). Recent evidence shows valence is integrated at judgment, with people averaging the mixed CS–US associations (Weber et al., 2025). Moreover, the varied US content in Experiment 1 (such as images of both people and animals—e.g., a fierce dog and a hurt kitten) could have activated more pronounced emotional responses than Experiment 2, which only had threatening faces.

Secondly, our data confirms that memory processes are central to EC (also see Bar-Anan et al., 2010; Gast, 2018). Across both experiments, the most consistent finding was that EC depends on contingency awareness and that how participants represented the CS–US relationship in memory predicted their later evaluations. Ambivalent CSs remembered as paired with negative USs were evaluated more negatively, and those remembered as paired with positive USs more positively. Ambivalent pairings resemble everyday social information, where a person or object is associated with both positive and negative cues. Our results suggest that people reconstruct the core of such mixed evidence, and that this reconstructed meaning, rather than mere co-occurrence, drives their evaluative response. Even though trait anxiety did not predict EC, the mechanism we proposed, cognitive biases shaping evaluative outcomes, remains a plausible route through which attitudes are formed from ambiguous input.

Concerning the lack of trait anxiety effect, we believe that the attentional findings might provide an explanation. In Experiment 1, eye-tracking data revealed a strong structural position bias: participants looked more at the US element closest to the screen center, regardless of valence, and this structural position bias overshadowed any other bias. After redesigning the stimuli in Experiment 2, we were able to better capture potential anxiety-related attention biases, as the positioning now allowed for equal viewing opportunities for the US elements, with the central focus being the CS. Contrary to our expectations, experiential avoidance moderated the link between trait anxiety and attention, such that highly anxious and highly avoidant participants dwelled more on negative elements in ambivalent stimuli. Although this moderation effect was not a priori predicted and should therefore be interpreted with caution pending replication, it tentatively suggests that attentional bias during attitude formation is not a simple, general feature of trait anxiety. Rather, it may emerge when there is genuine competition for attention and when a motivational factor, such as avoidance of threat, is engaged. Anxiety-related cognitive biases thus appear to be context-dependent rather than globally expressed.

This interpretation helps situate our null results within the broader attitude literature. We deliberately used emotionally meaningful but not strongly self-relevant USs (faces with different expressions, people and animals in various contexts). Such stimuli afford experimental control but may not reliably activate the concerns that drive anxiety-related processing. It is plausible that individual differences in EC-based attitude change would be more pronounced when USs are personally relevant (e.g., social-evaluative cues for socially anxious individuals, contamination images for health-anxious individuals) or when state anxiety is experimentally induced before conditioning. In those contexts, attention and memory are more likely to prioritize negative information and thus to shape the evaluative representation that emerges from mixed-valence input.

We also acknowledge the limits of our data. Although the present experiments were adequately powered, the moderation and mediation effects were very small, and some pathways may therefore have gone undetected. More importantly, the associations between EC and the personality variables we attempted to mediate were themselves close to zero. Prior studies detecting such effects relied on substantially larger samples (e.g., Vogel, Hütter, & Gebauer, 2019 (*N* = 576)). This pattern aligns with the mixed evidence in the literature (e.g., Casini et al., 2023; Vogel, Hütter, & Gebauer, 2019; Bunghez et al., 2023; Lazar et al., 2025) and suggests that the relation between negative affectivity and EC-based attitudes is small and likely context-dependent. When the basic trait–attitude association is weak, even well-specified mediation models are unlikely to succeed. For future work in attitude and social cognition, it may be more productive to increase the relevance and arousal of the USs, manipulate state anxiety, and match the threat domain of the USs to the participants’ dominant concerns. Under those circumstances, cognitive biases should be more strongly expressed and better positioned to influence evaluative learning.

In sum, our findings do not support a simple trait-driven account in which anxious or neurotic individuals invariably acquire more negative conditioned attitudes because they chronically attend more to negative information, remember it better, and interpret it more negatively. Instead, they point to a more nuanced picture: EC is robust under clear conditions and largely determined by what people encode and remember about the pairing; cognitive biases can shape that encoding, but primarily when evaluative information is ambiguous, personally relevant, or processed under anxiety; and personality differences in EC are most likely to emerge under those circumstances, not in neutral, highly controlled learning tasks. From an attitude and social-cognition perspective, this shifts the focus from null results to specifying the conditions under which anxiety-related cognitive biases are likely to affect evaluative conditioning and, more broadly, how people form and update their attitudes in the face of mixed-valence information.

## Additional File

The additional file for this article can be found as follows:

10.5334/irsp.1205.s1 Supplemental Materials. Additional figures and tables for both experiments, detailed exploratory analyses and results, along with a summary table outlining the findings for each experiment in relation to the tested hypotheses.

## Data Availability

The related pre-registrations are available at: https://aspredicted.org/n3wv-sp5h.pdf and https://osf.io/azynk/?view_only=1f25dbade0f94f6db65f0be61d2abcd0, while the dataset and R script are available at: https://osf.io/7kj9y/?view_only=472899933f524e7b835f939bf1aded5f.

## Footnotes

We use the term threat-related/threatening information or stimuli in line with the terminology adopted by the cited authors, referring broadly to negative information or stimuli.
