# Supplementary material for: Anxiety, Cognitive Biases, and Evaluative Conditioning: An Eye-Tracking Experiment
Source: Int Rev Soc Psychol. 2026 May 11;39:7. doi: 10.5334/irsp.1205 (PMC13178600; doi:10.5334/irsp.1205)
Supplement: Supplemental Materials. — Additional figures and tables for both experiments, detailed exploratory analyses and results, along with a summary table outlining the findings for each experiment in relation to the tested hypotheses. [file irsp-39-1205-s1.pdf]

Supplemental Materials

Anxiety, Cognitive Biases, and Evaluative Conditioning:

An Eye-Tracking Experiment using Ambivalent Stimuli

EXPERIMENT 1

Tables

Table S1.1

Descriptives Statistics for Variables Used in Analyses

Variable	Mean	SD	Min	Max	Skewness	Kurtosis
Neuroticism	132.23	27.98	64	218	-0.04	-0.18
- anxiety	23.61	6	10	40	0.15	-0.31
- fury	21.04	6.28	9	35	0.16	-0.84
- depression	21.91	6.48	8	40	0.28	-0.37
- timidity	22.49	5.92	8	40	0.08	-0.52
- impulsivity	23.54	5.14	11	39	-0.04	-0.11
- vulnerability	19.64	5.8	8	34	0.04	-0.52
CS pre-rating (amb)	5.66	1.42	1.5	8.5	-0.32	-0.31
CS post-rating (amb)	5.18	1.51	1	8.5	-0.28	-0.13
CS pre-rating (neg)	5.58	1.63	1	9	-0.3	-0.38
CS post-rating (neg)	4.45	1.94	1	8.5	-0.02	-0.77
CS pre-rating (pos)	5.66	1.69	1.5	9	-0.23	-0.53
CS post-rating (pos)	6.14	1.83	1	9	-0.46	-0.19
ECmonoPRE	0.08	1.98	-5.5	6	-0.12	-0.19
ECmonoPOST	1.69	2.83	-5.5	8	0.14	-0.32
Net change (amb)	-0.48	1.34	-5.25	3.5	-0.77	1.82
Contingency Awareness	4.15	1.99	0	8	0.06	-0.78
Memory Bias	0	1.25	-4	4	0.01	0.32
Interpretation Bias	24.97	5.97	12	48	0.74	0.59
Dwell time CS	1227.56	423.13	27.88	2410.88	0	-0.12
Dwell time US	1544.93	403.8	381.34	2760.22	0.31	0.37
Dwell time on + within amb	809.1	227.74	195.41	2440.53	1.68	9.36
Dwell time on - within amb	827.49	223.08	240.66	1602.5	0.46	1.06
First AOI accessed	0.23	3.51	-14	12	-0.12	1.95

Table S1.2

Mixed Analysis of Variance Results

	Effect	*df*	*F*	*P*
1	(Intercept)	273	7451.748	.000
2	US valence	499	37.82	.000
3	Time	273	36.704	.000
4	US valence x Time	445	58.624	.000

Note. Significance levels: ***(*p* <.001) , **(*p* <.01), *(*p* <.05), *ns* (not significant).

Table S1.3

Pairwise Comparisons over Time by US Condition

	US Valence	Group1	Group2	Value	df	p	
1	amb	pre	post	5.9	273	.000	***
2	neg	pre	post	9	273	.000	***
3	pos	pre	post	-4.42	273	.000	***

Note. Significance levels: ***(*p* <.001) , **(*p* <.01), *(*p* <.05), *ns* (not significant).

Table S1.4

Pairwise Comparisons between US Conditions

	Time	Group1	Group2	Value	*df*	*p*	
1	pre	amb	neg	0.7	273	.488	ns
2	pre	amb	pos	-0.02	273	.980	ns
3	pre	neg	pos	-0.64	273	.523	ns
4	post	amb	neg	6.32	273	.000	***
5	post	amb	pos	-7.68	273	.000	***
6	post	neg	pos	-9.9	273	.000	***

Note. Significance levels: ***(*p* <.001) , **(*p* <.01), *(*p* <.05), *ns* (not significant).

Table S1.5

Mixed Model Testing the Moderation Effect of Anxiety on the EC Standard Effect (pos vs neg)

Random Effects

**Groups**		**Variance**	**Std. Dev.**
**ID**	(Intercept)	0.25	0.50
	CS pre-evaluations	0.01	0.09
	US valence (pos vs neg)	4.27	2.07
**CS**	(Intercept)	0.03	0.16
	US valence x Anxiety	0.00	0.03
**Residual**		3.21	1.79

Number of observations: 2192, groups: id, 274; CS, 8

Fixed Effects

	**Estimate**	**Std. Err**	**95% CI**	***df***	***t Value***	***p***	
(Intercept)	2.71	0.13	2.45 – 2.96	69	20.97	<0.001	***
CS pre-evaluations	0.45	0.02	0.41 – 0.49	1351	22.83	<0.001	***
US Valence	1.67	0.17	1.34 – 1.99	272	10.06	<0.001	***
Anxiety	-0.01	0.01	-0.03 – 0.01	257	-1.11	0.268	
US valence x Anxiety	0.04	0.03	-0.02 – 0.10	23	1.42	0.168	

Note. Significance levels: ***(p <.001) , **(p <.01), *(p <.05), *ns* (not significant).

US valence was coded as follows: positive condition =”+0.5” and negative condition =”-0.5"

Exploratory Analysis Related to Monovalent Conditions

Nevertheless, to maximize the value of our collected eye-tracking data, despite the position bias within ambivalent stimuli, we decided to run an alternative model. Considering the analysis above on the strong impact of position, ambivalent stimuli could be regarded as generally positive or negative US (depending on which valence is closer to the center) while accompanied by a peripheral element of opposite valence. This alternative view for the ambivalent stimuli allows for comparison in effects against purely positive or negative US that have the peripheral element of the same valence. We calculated the monovalent (difference in post conditioning CS ratings that were paired with positive versus those with negative US conditions) and ambivalent (difference in post-conditioning CS ratings that were paired with ambivalent stimuli with positive versus those with negative elements in the center) while controlling for the pre-conditioning ratings.

Using a linear regression model, we investigated whether there is a significant difference in effects obtained using USs of pure valence over those that contain an element of opposite valence. Results in Table S1.5 indicate there is a significant moderate to high difference in effects with Cohen $d = 0.48$, 95% CI [0.36; 0.61], SE = 0.19, t = 7.93, $p < .001$ which means that stimuli of pure valence were much more effective than those accompanied by opposite valence elements.

Investigating whether this difference is mainly due to the positive or negative conditions, results indicate a slightly larger difference for the positive condition (Table S1.6), with a Cohen $d = 0.39$, 95% CI [0.26; 0.51], SE = 0.14, t = 6.38, $p < .001$ than the negative condition (Table S1.7), with a Cohen $d = -0.33$ 95% CI [0.36; 0.61], SE = 0.12, t = -5.45, $p < .001$.

Table S1.6

Linear Regression testing the difference in EC effects between monovalent and ambivalent , controlling for the pre-ratings.

Estimate	Estimate	SE	t-value	*p*	
POST Diff mono vs ambi EC	1.49	0.19	7.93	.000	***
PRE Diff mono vs ambi EC	0.36	0.07	5.17	.000	***

Note. The difference is calculated comparing monovalent EC (pos - neg) versus ambivalent EC (ambivalent US that have central positive elements - ones with negative)
Significance levels: ***(*p* <.001) , **(*p* <.01), *(*p* <.05), ns (not significant).

Table S1.7

Linear Regression assessing the effect of purely positive US versus those with central positive elements accompanied by an opposite valence element, while controlling for the pre-ratings

Estimate	Estimate	SE	t-value	*p*	
POST Diff mono vs ambi EC	0.87	0.14	6.38	.000	***
PRE Diff mono vs ambi EC	0.24	0.06	3.64	.000	***

Note. Significance levels: ***(*p* <.001) , **(*p* <.01), *(*p* <.05), *ns* (not significant).

Table S1.8

Linear Regression evaluating the effect of purely negative US versus those with central negative elements accompanied by an opposite valence element, while controlling for the pre-ratings

Estimate		SE	t-value	*p*	
POST Diff mono vs ambi EC	-0.64	0.12	-5.45	.000	***

PRE	Diff	mono vs amb EC	0.17	0.06	3.08	.002	**

Note. Significance levels: ***(*p* <.001) , **(*p* <.01), *(*p* <.05), *ns* (not significant).

Figures

Figure S1.1

Parallel mediation model testing the effect of neuroticism and three mediators on CS ratings.

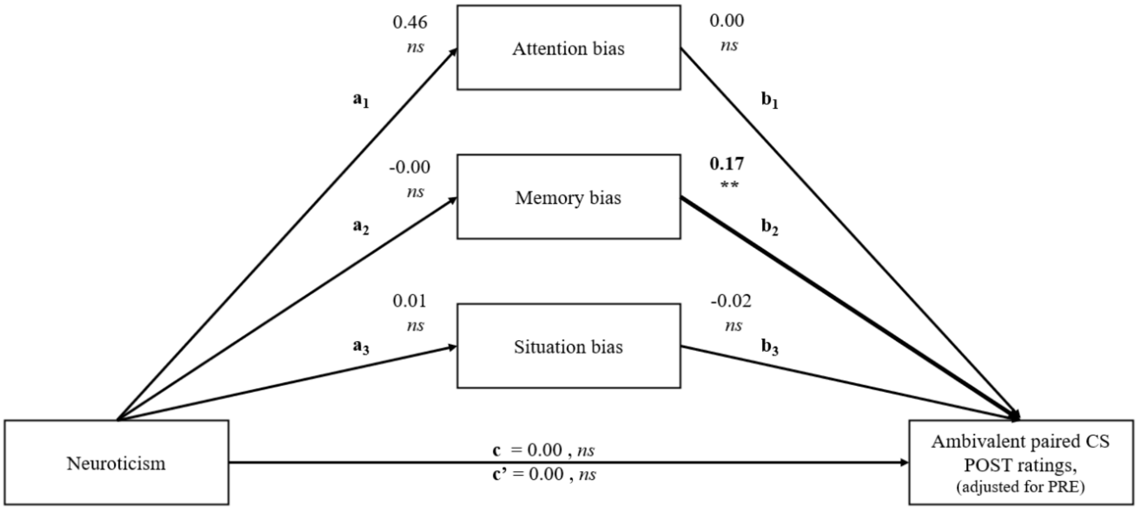

Note. The diagram presents the mediation paths a, b, c (total effect) and c' (direct effect). Significance levels: ***(*p* < .001) , **(*p* < .01), *(*p* < .05), *ns* (not significant).

Figure S1.2

Parallel mediation model testing the effect of anxiety and 3 mediators on ambivalent CS ratings.

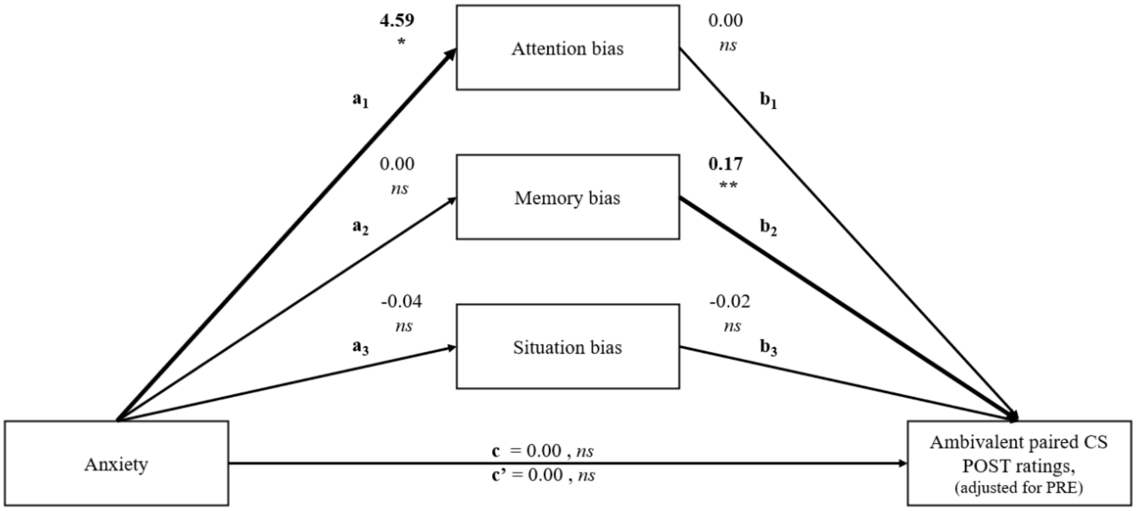

Note. The diagram presents the mediation paths a, b, c (total effect) and c' (direct effect)
Significance levels: ***(p < .001) , **(p < .01), *(p < .05), *ns* (not significant).

Mediation Models applying all pre-registered criteria.

Figure S1.3

Parallel mediation model testing the effect of neuroticism and three mediators on CS ratings.

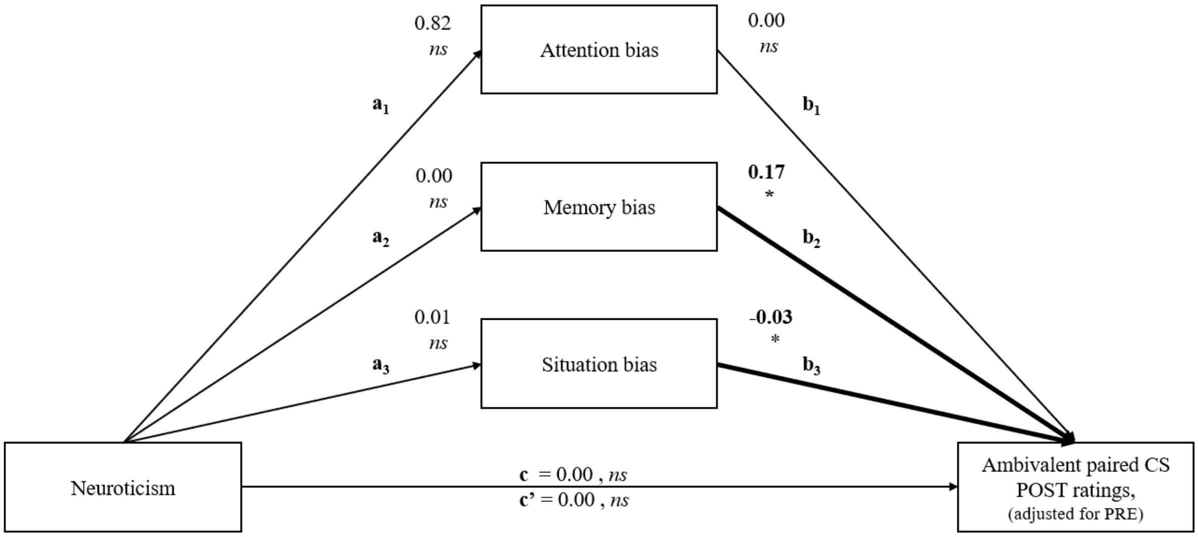

Note. The diagram presents the mediation paths a, b, c (total effect) and c' (direct effect).
Significance levels: ***(p < .001) , **(p < .01), *(p < .05), *ns* (not significant).

Figure S1.4

Parallel mediation model testing the effect of anxiety and three mediators on CS ratings.

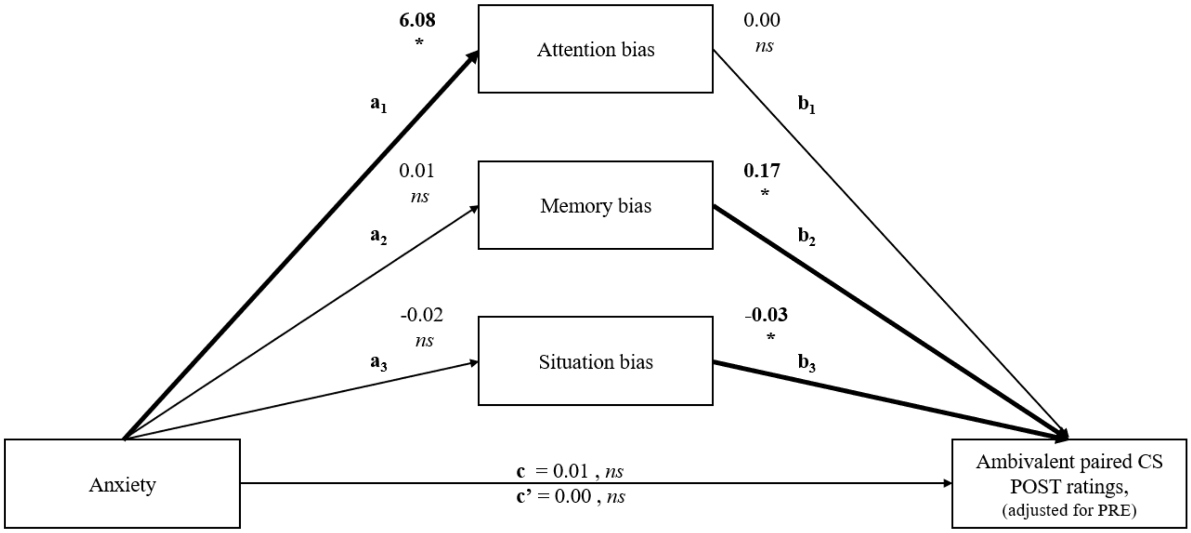

Note. The diagram presents the mediation paths a, b, c (total effect) and c' (direct effect). Significance levels: ***(p < .001) , **(p < .01), *(p < .05), *ns* (not significant).

EXPERIMENT 2
Tables

Table S2.1

Descriptives Statistics for Variables Used in Analyses.

Variable	Mean	SD	Min	Max	Skewness	Kurtosis
ANXc	0	8.24	-20.86	19.14	-0.19	-0.65
BISc	0	3.95	-11.67	8.33	-0.33	-0.15
EAc	0	11.8	-32.11	32.89	0.07	-0.22
ambPRE	5.39	1.44	1.75	8.75	-0.16	-0.61
ambPOST	5.42	1.38	1.75	8.5	-0.2	-0.5
negPRE	5.48	1.8	1	9	-0.18	-0.68
negPOST	5.23	1.85	1	9	-0.12	-0.71
posPRE	5.38	1.71	1.5	9	-0.1	-0.7
posPOST	5.73	1.67	1	9	-0.39	-0.18
ECmonoPRE	-0.1	1.94	-5	4.5	-0.07	-0.31
ECmonoPOST	0.51	2.28	-5	8	0.18	0.11
ambDIFF	0.03	1.3	-3.75	5.5	0.25	1.16
totVA	4.04	1.79	0	8	0.15	-0.66
mmb	-0.07	1.26	-3	4	0.2	0.04
dovCSavg	1499.96	452.21	230.66	2712.38	0.28	-0.25
dovUStot	1662.94	445.83	511.06	2968.62	-0.12	-0.21
dovUShappyavg	874.8	217.9	313.66	1541.62	0.01	0.22
dovUSangryavg	864.9	240.75	226.31	1631.91	-0.05	0.06
DOVhappyM	1663.78	441.8	577.5	2966.31	-0.11	-0.23
DOVangryM	1662.1	469.98	414.12	2970.94	-0.13	-0.25
DOVrap	-0.05	0.28	-0.86	0.68	0.21	-0.29
DOVnet	9.91	169.2	-952.66	681.62	-0.6	6.82
DOVnetM	1.68	192.57	-649.75	710.94	-0.02	0.74
firstAOI	0.83	4.44	-14	14	-0.07	0.62

Table S2.2

Mixed Analysis of Variance Results

	Effect	*df*	*F*	*p*	
1	(Intercept)	293	6456.383	.000	***
2	US valence	513	2.81	.069	
3	Time	293	0.5	.482	
4	US valence x Time	540	13.42	.000	***

Note. Significance levels: ***(*p* <.001) , **(*p* <.01), *(*p* <.05), *ns* (not significant).

Table S2.3

Pairwise comparisons over time by US condition

	US Valence	Group1	Group2	Value	df	p	
1	amb	pre	post	-0.37	293	.711	ns
2	neg	pre	post	2.51	293	.013	*
3	pos	pre	post	-3.73	293	.000	***

Note. Significance levels: ***(*p* <.001) , **(*p* <.01), *(*p* <.05), *ns* (not significant).

Table S2.4

Pairwise comparisons between US conditions

	Time	Group1	Group2	Value	df	p	
1	pre	amb	neg	-0.83	293	.408	ns
2	pre	amb	pos	0.13	293	.896	ns
3	pre	neg	pos	0.84	293	.401	ns
4	post	amb	neg	1.97	293	.050	*
5	post	amb	pos	-3.18	293	.002	**
6	post	neg	pos	-3.8	293	.000	***

Note. Significance levels: ***(*p* <.001) , **(*p* <.01), *(*p* <.05), *ns* (not significant).

Table S2.5

Mixed Model Testing the Moderation Effect of Anxiety on the EC Standard Effect (pos vs neg)

Random Effects

**Groups**		**Variance**	**Std. Dev.**
**ID**	(Intercept)	0.42	0.65
	CS pre-evaluations	0.00	0.06
	US valence (pos vs neg)	1.12	1.06
**CS**	(Intercept)	0.03	0.18
	CS pre-evaluations	0.00	0.03
	US valence x Anxiety	0.00	0.03
**Residual**		2.96	1.72

Number of observations: 2352, groups: id, 294; CS, 8

Fixed Effects

	**Estimate**	**Std. Err**	**95% CI**	***df***	***t Value***	***p***	
(Intercept)	3.10	0.13	2.85 – 3.35	28	24.40	<0.001	***
CS pre-evaluations	0.43	0.02	0.39 – 0.47	24	19.70	<0.001	***
US Valence	0.54	0.12	0.31 – 0.77	291	4.57	<0.001	***
Anxiety	0.00	0.01	-0.02 – 0.01	278	-0.32	0.749	
US valence x Anxiety	0.02	0.02	-0.02 – 0.05	8	0.87	0.407	

Note. Significance levels: ***(*p* <.001) , **(*p* <.01), *(*p* <.05), *ns* (not significant).

US valence was coded as follows: positive condition =”+0.5” and negative condition =”-0.5"

Figures

Figure S2.1

Moderated Parallel Mediation Model for Ambivalent paired CS Evaluations & Anxiety

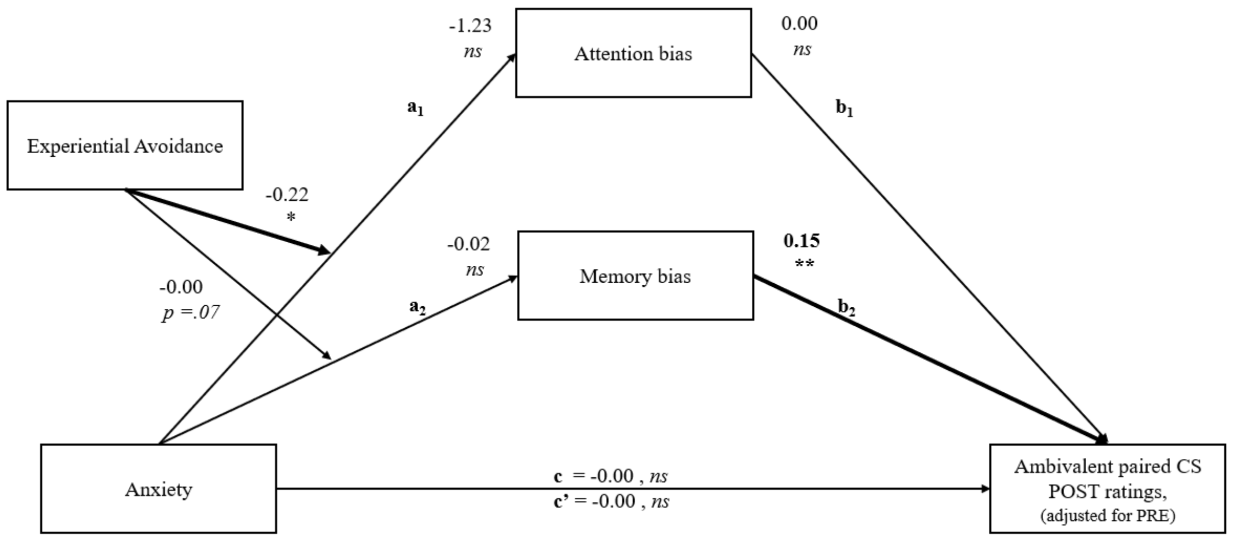

Note: Moderator: W = Experiential Avoidance; Mediators: M1 = attention bias & M2 = memory bias. The diagram presents the two mediation paths a and b, c (total effect) and c' (direct effect). Significance levels: ***($p < .001$) , **($p < .01$), *($p < .05$), *ns* (not significant).

Figure S2.2

Simple Slope Analysis of the Moderation Effect of Experiential Avoidance on the Interaction between Trait Anxiety and Biases

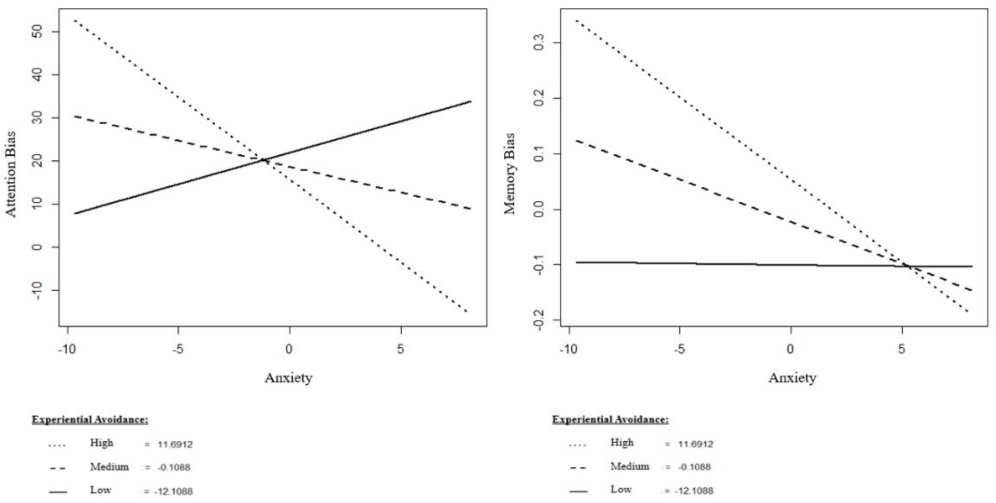

Figure S2.3

Moderated Parallel Mediation Model for Ambivalent Paired CS Evaluations & BIS

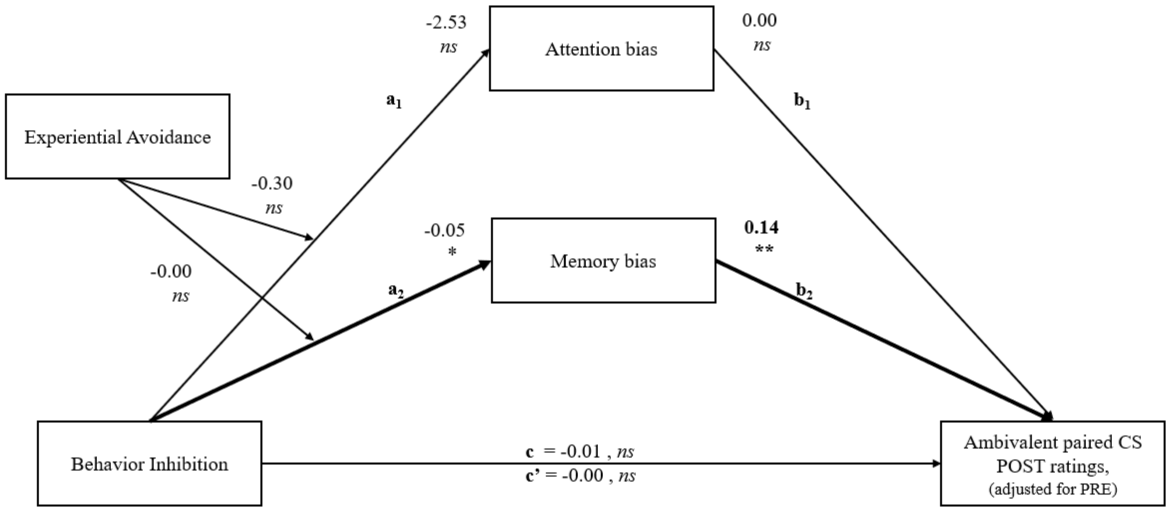

Note: Moderator: W=Experiential Avoidance; Mediators: M1=attention bias & M2=memory bias
The diagram presents the mediation paths a and b, c (total effect) and c' (direct effect).
Significance levels: ***(*p* < .001), **(*p* < .01), *(*p* < .05), *ns* (not significant).

Mediation Models applying all pre-registered criteria.
Figure S2.4

Moderated Parallel Mediation Model for Ambivalent paired CS Evaluations & Anxiety (using all pre-registered exclusion criteria)

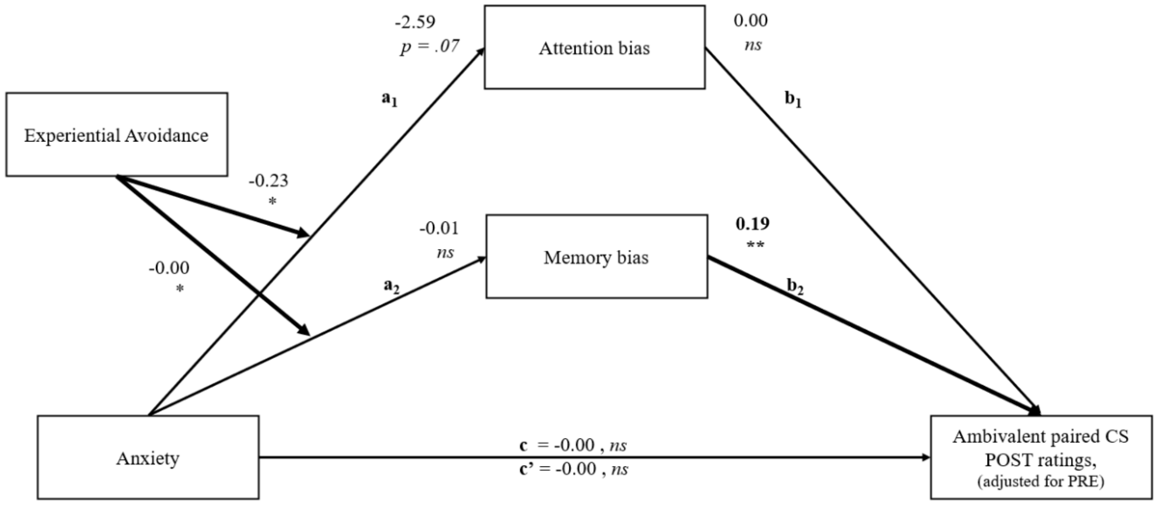

Note: Moderator: W=Experiential Avoidance; Mediators: M1=attention bias & M2=memory bias
The diagram presents the mediation paths a and b, c (total effect) and c' (direct effect).
Significance levels: ***(*p* < .001) , **(*p* < .01), *(*p* < .05), *ns* (not significant).

Figure S2.5

Simple Slope Analysis of the Moderation Effect of Experiential Avoidance on the Interaction between Trait Anxiety and Biases (using all pre-registered exclusion criteria)

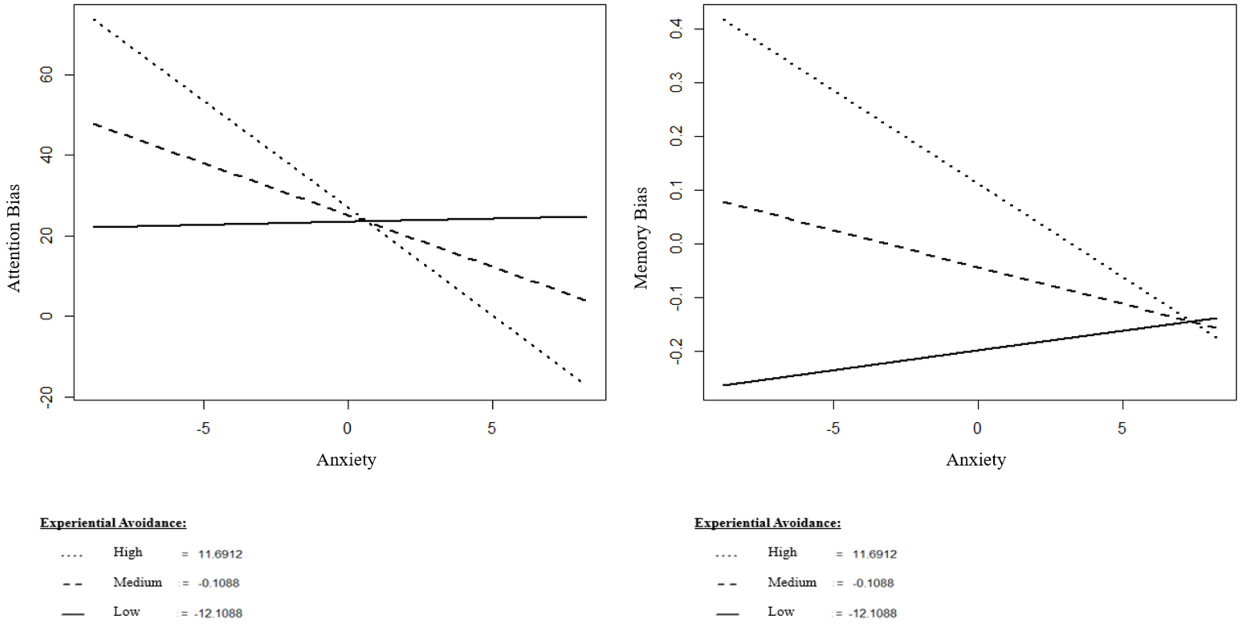

Figure S2.6

Moderated Parallel Mediation Model for Ambivalent paired CS Ratings & Behavior Inhibition (using all pre-registered exclusion criteria)

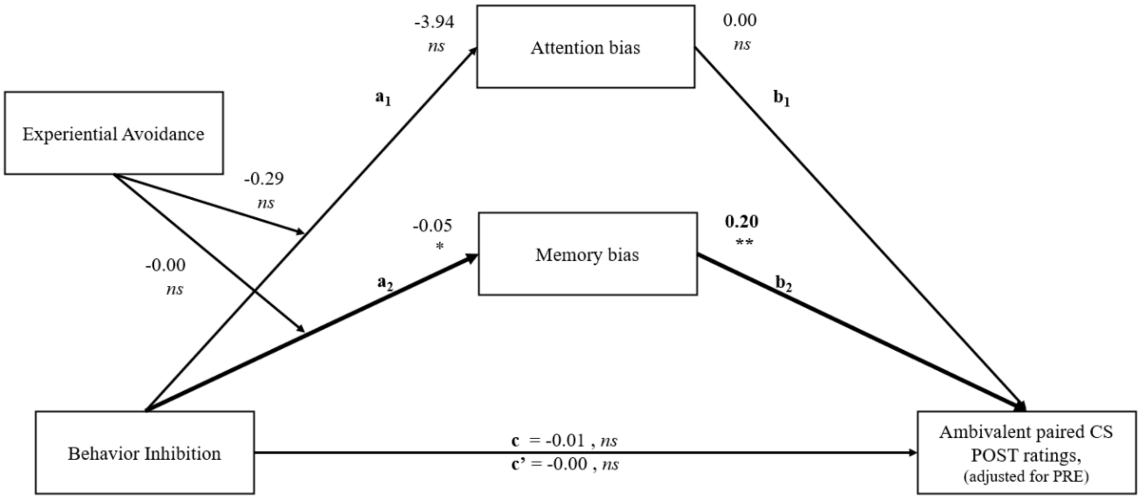

Note: Moderator: W=Experiential Avoidance; Mediators: M1=attention bias & M2=memory bias
The diagram presents the mediation paths a and b, c (total effect) and c' (direct effect).
Significance levels: ***(p < .001) , **(p < .01), *(p < .05), *ns* (not significant).

GENERAL SUMMARY

Table S3

General Summary of Results from both Experiment 1 and Experiment 2.

Hypothesis	Measure & Analysis	Results Exp 1	Results Exp 2
**H1** Significant standard EC effects (positive vs negative)	CS evaluations<br>MIXED ANOVA	$d = 0.60$, 95% *CI* [0.47, 0.73], $p < .001$	$d = 0.22$, 95% *CI* [0.11, 0.34], $p < .001$)
**H2** Change in time of ambivalent paired CSs correlate with personality (neuroticism / anxiety / behavior inhibition)	NEOPIR/IPIP/BIS<br>& CS evaluations CORRELATION MATRIX	neuroticism<br>($r = 0.11, p = .075$)<br>anxiety facet<br>($r = 0.07, p = .262$)	anxiety facet<br>($r = -0.00, p = .999$)<br>BIS<br>($r = 0.02, p = .781$)
**H3.a** Individuals high in anxiety / neuroticism / BIS present a negative attention bias by looking more at negative elements of ambivalent US which leads to a more negative evaluation of paired CS	Duration of Visits in ms within each side of ambivalent US<br>(pos-neg)<br>PROCESS model 4 (exp 1) & 7 (exp 2)	Neuroticism:<br>$a_1 = 0.457, p = .359$<br>Anxiety:<br>$\mathbf{a_1 = 4.59, p = .046}$<br>$b_1 = 0.00, p = .432$	Anxiety:<br>$a_1 = -1.23, p = .350$<br>BIS:<br>$a_1 = -2.53, p = .358$<br>$b_1 = 0.00, p = .152$
**H3.b** Individuals high in anxiety / neuroticism / BIS show a negative memory bias by recalling the CSs paired with ambivalent USs as negative rather than positive thus leading to a more negative evaluation of paired CS	Number of ambivalent paired CSs that were incorrectly recalled as being paired with positive vs negative US<br>PROCESS model 4 (exp 1) & 7 (exp 2)	Neuroticism:<br>$a_2 = -0.00, p = .691$<br>Anxiety:<br>$a_2 = 0.00, p = .794$<br>$\mathbf{b_2 = 0.17, p = .004}$	Anxiety:<br>$a_2 = -0.02, p = .118$<br>BIS:<br>$\mathbf{a_2 = -0.05, p = .023}$<br>$\mathbf{b_2 = 0.15, p = .005}$
**H3.c** Individuals high in anxiety / neuroticism interpret the EC experiment situation as more negative which leads to a more negative evaluation of the CS	Ziegler Situation 5 taxonomy<br>PROCESS model 4	Neuroticism:<br>$a_3 = 0.01, p = .652$<br>Anxiety:<br>$a_3 = -0.04, p = .466$<br>$b_3 = -0.02, p = .177$	-
**H4** Individuals high in trait anxiety and also in experiential avoidance focus their attention and recall the positive elements within ambivalent US	BEAQ -experiential avoidance<br>PROCESS moderated mediation model 7	-	*Anxiety:*<br>$\mathbf{R^2 = .02, p = .016,}$<br>$\mathbf{F(1,289) = 5.82}$
